# Body composition and physical fitness in adults born small for gestational age at term: a prospective cohort study

**DOI:** 10.1038/s41598-023-30371-y

**Published:** 2023-03-01

**Authors:** Maria Matre, Cathrin Vano Mehl, Silje Dahl Benum, Laura Jussinniemi, Eero Kajantie, Kari Anne I. Evensen

**Affiliations:** 1 Department of Rehabilitation Science and Health Technology, Oslo Metropolitan University, Oslo, Norway; 2 Department of Clinical and Molecular Medicine, Norwegian University of Science and Technology, Trondheim, Norway; 3 Public Health Unit, Finnish Institute for Health and Welfare, Helsinki and Oulu, Finland; 4 Clinical Medicine Research Unit, Oulu University Hospital and University of Oulu, Oulu, Finland; 5 Children’s Hospital, Helsinki University Hospital and University of Helsinki, Helsinki, Finland; 6 Unit for Physiotherapy Services, Trondheim Municipality, Trondheim, Norway; 7 Children’s Clinic, St. Olavs Hospital, Trondheim University Hospital, Trondheim, Norway

**Keywords:** Medical research, Epidemiology

## Abstract

There is lack of research on body composition and physical fitness in individuals born small for gestational age (SGA) at term entering mid-adulthood. We aimed to investigate these outcomes in adults born SGA at term. This population-based cohort study included 46 adults born SGA with birth weight < 10th percentile at term (gestational age ≥ 37 weeks) (22 women, 24 men) and 61 adults born at term with birth weight ≥ 10th percentile (35 women, 26 men) at 32 years. Body composition was examined anthropometrically and by 8-polar bioelectrical impedance analysis (Seca® mBCA 515). Fitness was measured by maximal isometric grip strength by a Jamar hand dynamometer, 40-s modified push-up test and 4-min submaximal step test. Participants born SGA were shorter than controls, but other anthropometric measures did not differ between the groups. Men born SGA had 4.8 kg lower grip strength in both dominant (95% CI 0.6 to 9.0) and non-dominant (95% CI 0.4 to 9.2) hand compared with controls. Grip strength differences were partly mediated by height. In conclusion, body composition and physical fitness were similar in adults born SGA and non-SGA at term. Our finding of reduced grip strength in men born SGA may warrant further investigation.

## Introduction

Individuals born small for gestational age (SGA) are often defined as having a birth weight below the 10th percentile for their gestational age^1^. SGA is the most frequently used indicator of intrauterine growth restriction, which is a state where the foetus does not reach its genetic growth potential^2^. Males may be more susceptible to growth restriction in utero than females^3^. Being born SGA involves an extra vulnerability for later diseases, such as metabolic syndrome and cardiovascular diseases^4,5^. The prevalence of SGA depends on the birth weight standards used. When standards that are based on actual birth weights in the given population are used, 10% of term-born infants are by definition born SGA. In low- and middle-income countries the prevalence is around 20% according to Intergrowth standards^6^. Hence, the high prevalence represents a major concern for public health.

Most infants born SGA show spontaneous catch-up growth by two years of age, however approximately 10% do not, and persistent short stature is therefore one of the most common complications after being born SGA^4^. During childhood, studies have reported that those born SGA remain shorter and thinner with lower body mass index (BMI) than controls^7–9^, while there are reports of both less body fat^7^ and higher central adiposity^8^. In adulthood, studies have found individuals born SGA to be shorter^10–12^ and lighter^11,12^ than controls, but their BMI did not differ at 18^11^ or 22 years of age^12^. At 26 years of age, we did not find group differences in body composition, but women born SGA displayed lower lean mass than controls^13^. Another study indicated progression of adiposity from 22 to 30 years, as adults born SGA had more body fat, and their waist circumference increased, but there was no interaction effect with sex^12^. Also, low birth weight has been linked to reduced muscle mass and reduced muscle strength in childhood and adulthood^14^. A recent meta-analysis found a positive association between SGA and overweight and/or obesity^15^, whereas there were inconsistent findings of this association by sex^15^.

Muscular and cardiorespiratory fitness are two important health-related components of physical fitness^16^, that both have been reported to be associated with all-cause mortality, as well as non-communicable diseases^17,18^. Grip strength, a simple and widely used measure of muscular strength, has proven to be particularly relevant as it is a strong predictor of future physical function, morbidity, and mortality^18–20^. Several studies have found associations of birth weight with muscular and cardiorespiratory fitness in adulthood^21–23^. However, these studies have either included adults born preterm or not specified the gestational age of their participants, making it difficult to distinguish if the result is due to factors related to preterm birth or low birth weight. Three relatively small studies found no difference in cardiorespiratory fitness, measured by maximal oxygen uptake (VO_2max_), between term-born young adults with a birth weight at or below the 10th percentile and a control group^11,24,25^. On the other hand, two recent Swedish registry studies that included a large number of 18-year-old men born at term, found strong associations of birth weight with grip strength and cardiorespiratory fitness^26,27^.

There is lack of research on body composition and physical fitness in individuals born SGA entering mid-adulthood, an age when the prevalence of many non-communicable diseases starts to increase^28^. The aim of this study was to examine whether body composition and physical fitness differed between adults born SGA and non-SGA at term. We hypothesised that adults born SGA at term would display a less favourable body composition and lower level of fitness than the term-born control group.

## Methods

### Study design

This study is a part of the NTNU Low Birth Weight in a Lifetime Perspective study. The present study included two groups of adults born in 1986–1988; one group born SGA at term, and one group born non-SGA at term with birth weight ≥ 10th percentile, which serves as a control group. The participants took part in a larger data collection at 32 years of age. In addition to physical fitness tests, examinations included anthropometric measurements, examination of lung function, visual function as well as fine and gross motor function. Assessments were carried out from September 2019 to October 2020.

### Participants

**Figure 1:**
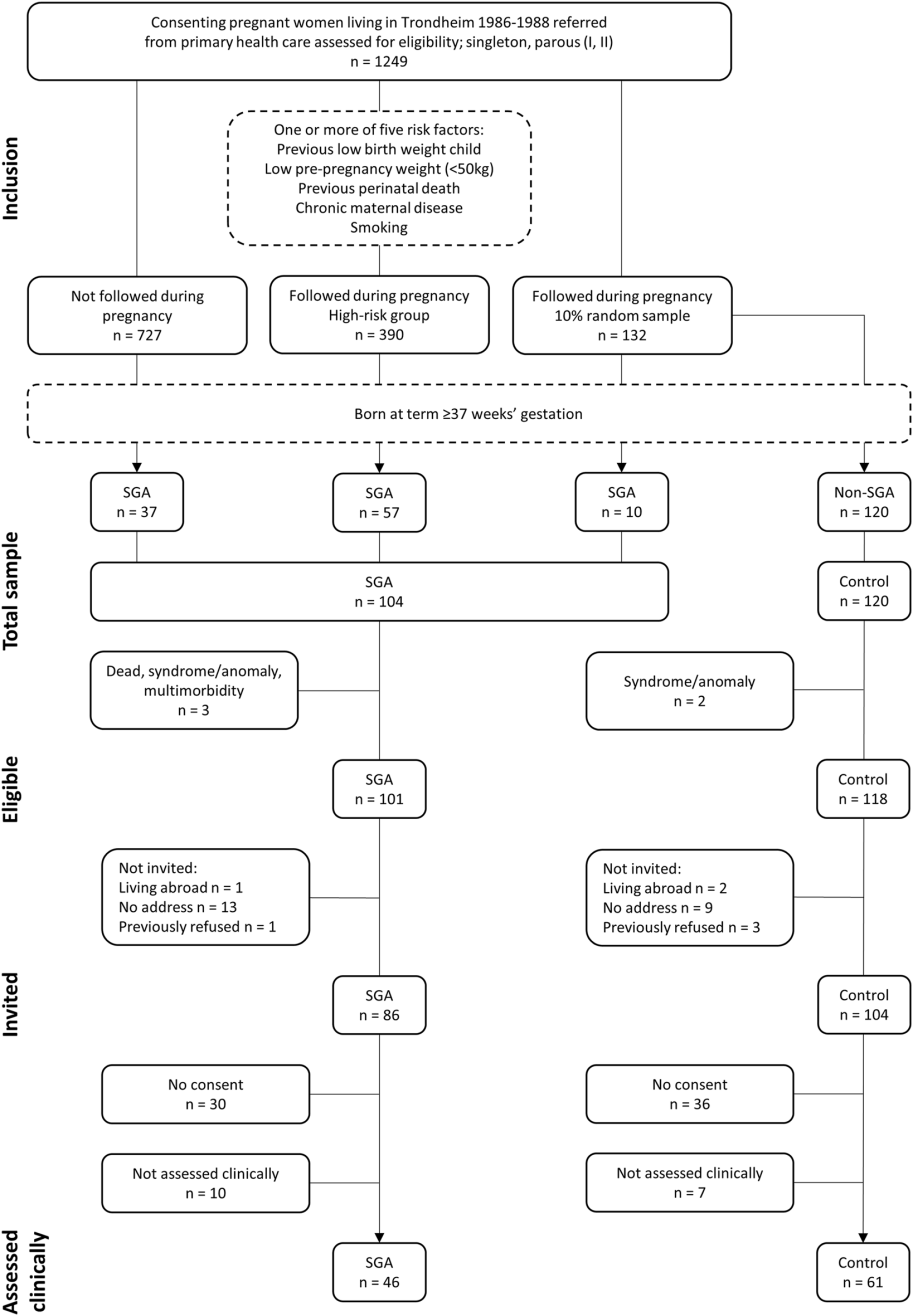
Flow of participants. *SGA* small for gestational age.

Participants were initially included in a multicentre study investigating the aetiology and consequences of intrauterine growth restriction^29,30^. Pregnant women living in the Trondheim region were enrolled before week 20 of pregnancy based on referral from general practitioners and obstetricians. Women were eligible if they had a singleton pregnancy and had been pregnant one or two times before (n = 1249). A 10% random sample of these women were selected to serve as a control group (n = 132), using a sealed envelope method. A group of women at high risk of giving birth to an SGA infant were selected for follow-up if they had one or more defined risk criteria for SGA birth; a previous low birth weight child, low pre-pregnancy weight (< 50 kg), previous perinatal death, presence of chronic maternal disease (chronic renal disease, essential hypertension, or heart disease), or maternal cigarette smoking at conception (n = 390). Women in the control group and the high-risk group were thoroughly followed during pregnancy and their infants were examined at birth. The rest of the women (n = 727) were not followed during pregnancy (Fig. 1).

At birth, all SGA infants born to mothers in either group were included in the SGA group (Fig. 1). An infant was defined as being born SGA if the birth weight was < 10th percentile for gestational age (GA), corrected for sex and parity, according to a reference standard using data from the Norwegian Medical Birth Registry^29^. Non-SGA infants born to mothers in the random sample were included in the control group. They were born with a birth weight ≥ 10th percentile. GA was based on the first day of the mother’s last menstrual period if this was accurately recalled ± 3 days. Ultrasound based GA was used if the last menstrual period was not recalled, or if there was a discrepancy of more than 14 days. Both groups were born at term (GA ≥ 37 weeks)^29,30^.

The total sample included 104 participants born SGA and 120 controls (Fig. 1). Three individuals born SGA and two controls were excluded due to death, congenital syndrome/anomaly, or multimorbidity. Of the eligible, 15 individuals born SGA and 14 controls were not invited because they were living abroad, had no contact information or had previously refused to participate. Thus, a total of 190 were invited to the present study, 86 in the SGA group and 104 in the control group. Of these, 30 individuals born SGA and 36 controls did not consent to participate. Furthermore, 10 individuals born SGA and seven controls were not assessed clinically. Thus, 46 participants born SGA and 61 controls were assessed clinically, corresponding to 56.3% of the invited.

### Non-participants

There were no significant differences between participants and those who did not consent or were not assessed clinically regarding sex, gestational age, birth weight, head circumference, body length, ponderal index, maternal age at child’s birth or parental socioeconomic status (SES) in either group (data not shown). From the 26-year follow-up data were available on height, weight, BMI, waist and hip circumference, skinfold thickness and body composition measured by dual-energy x-ray absorptiometry (DXA). In the SGA group there were no differences, but in the control group, participants weighed 8.9 (95% CI 1.0 to 16.8) kg less than those who did not consent or were not assessed clinically.

### Background characteristics

At birth, the infants in both groups were weighed to the nearest 10 g on a standard scale, and crown-heel length was measured with both legs extended to the nearest mm^30^. Ponderal index (g/cm^3^) was calculated based on these measurements.

Parental socioeconomic status (SES) was calculated when participants attended the 14-year follow-up, supplemented for two participants at the 19-year follow-up, according to Hollingshead’s Two Factor Index of Social Position^31^, based on the parents’ education and occupation. This gives a social class rating from 1 (lowest) to 5 (highest).

Educational attainment at the 32-year follow-up was collected by self-report and classified according to the International Standard Classification of Education (ISCED) levels 1 through 8. These were recoded into three categories: Lower secondary education or lower (ISCED levels 1–2) as no more than 10th class level, intermediate education (ISCED levels 3–5) as 11th–14th class level, and lower tertiary education or higher (ISCED levels 6–8) as a bachelor’s degree or higher.

### Outcome measures

Assessments were carried out at NTNU/St. Olavs Hospital in Trondheim, Norway. A brief medical interview was conducted prior to examination, including whether the participant was pregnant, had a musculoskeletal diagnosis or other conditions affecting physical functioning. If the participant had a condition that made them unable to perform a physical test or that could be worsened by testing, they did not perform that particular test. All examinations were carried out by experienced and specially trained examiners, blinded to birth weight group. Anthropometric measurements were performed by a nurse and physical fitness tests by two physiotherapists and a medical research student. The examinations were carried out in the same order for each participant.

At follow-up, the participants’ height, waist and hip circumference were measured to the nearest mm. Waist circumference was measured at the mid-point between the lowest rib and the crista iliaca, and hip circumference at the maximal circumference over the buttocks. Weight was measured by bioelectric impedance analysis using a Seca medical Body Composition Analyzer (Seca® mBCA 515) with a 100 g accuracy. Body mass index (BMI, kg/m^2^) and waist-to-hip ratio (waist circumference/hip circumference) was calculated. Bioelectrical impedance analysis measures included percent body fat, fat mass, fat free mass, skeletal muscle mass, total body water and extracellular water using the Seca 115 analytics software (Seca GmbH, Hamburg, Germany).

Muscular fitness was measured by the maximal isometric grip strength of the hands and forearm muscles. A Jamar (Smith and Nephew, Memphis, TN) hand dynamometer was used. The dynamometer has 5 handle positions; position 3 and 4 were used for women and men, respectively. The participants were seated during the test, with shoulder abducted, a 90° angle in the elbow and a neutral position in the wrist, without support of the forearm^32^. Measurement was repeated three times in both dominant and non-dominant hand with 30 s recovery in between each attempt. Grip strength was measured in kg force and the maximal grip strength of the three measurements for each hand was used in the analysis. One participant in the control group could not perform the grip strength test with the dominant hand due to a hand fracture.

The 40-s modified push-up test measures the muscular strength and endurance capacity of the upper body^33^ and is modified to improve standardisation. The participants started laying prone on a mat with their hands close to the shoulders and feet hip-width apart with their toes on the mat^33^. Before every push-up they had to clasp hands behind their back before pushing themselves to a straight leg push-up. In the top position they had to touch either of their hands with the other hand before returning to the push-up position and returning to the down-position. The number of correctly performed push-ups in 40 s were registered. One participant in the control group could not perform the push-up test due to a hand fracture.

The Åstrand-Ryhming step test is a 4-min submaximal step-test that measures cardiorespiratory fitness^34^. The participants stepped on and off the step for four minutes paced by a metronome set to 46 beats per minute (i.e., 23 times up on the step/min). The height of the step was adapted to sex: 33 cm for women and 40 cm for men. Heart rate was observed during the test using a heart rate monitor (Firstbeat Technologies Oy) and recorded after 4 min of stepping and after being seated for 2 min. Two participants born SGA were not able to complete the test and were excluded from the analysis.

### Statistical analysis

The analyses were conducted in SPSS version 27 (IBM Statistics). A p-value of less than 0.05 was considered statistically significant. Background characteristics were examined using Student’s t-test for continuous data, Exact Mann–Whitney U test for ordinal data and Pearson’s Chi square test for dichotomous variables. Group differences in outcome measures were analysed using independent samples t-test. The assumption of normally distributed variables was checked by visual inspection of histogram, boxplot, and Q–Q-plots of standardised residuals. As physical fitness differs between women and men^35,36^, we performed separate analyses by sex. Differences in physical fitness between groups were adjusted for height as a potential mediating factor in a univariate general linear model, since height has been consistently correlated with both being born SGA^4,12,37^ and physical fitness in previous literature^22^.

To investigate whether physical conditions affected the results, sensitivity analyses were performed by excluding participants who were pregnant, had a musculoskeletal diagnosis or other conditions affecting physical functioning, as reported by the participants in the brief medical interview.

A priori power calculations suggested, based on previous follow-up numbers in the SGA (n = 64) and control group (n = 81)^38^, that we would have the power to detect differences of 0.48 SD units with an alpha-level of 0.05 and a power of 80%, and 0.67 SD units with an alpha-level of 0.01 and desired power of 90%.

### Ethics

The study was approved by the Regional Committee for Medical and Health Research Ethics in Central Norway (23879). Written informed consent was obtained from all participants. All methods were performed in accordance with the relevant guidelines and regulations. The data was pseudonymised and stored securely on a remote server with a two-step identifier. All methods were non-invasive and entailed low risk for injury or adverse events. An appointed doctor was medically responsible during data collection. Participants in need of health services were referred as appropriate.

## Results

Participants’ background characteristics are shown in Table 1. There were 22 (47.8%) women in the SGA group and 35 (57.4%) in the control group (p = 0.327). Educational attainment did not differ between the groups (Table 1).

**Table 1:** Background characteristics of adults born small for gestational age (SGA) and non-SGA (control) at term.

	SGA (n = 46)		Control (n = 61)		p-value
	Mean	(SD)	Mean	(SD)	
Gestational age (weeks)	39.6	(1.2)	39.8	(1.2)	0.351
Birth weight (g)	2918	(216)	3686	(467)	< 0.001
Birth weight SD score	-1.5	(0.4)	0.2	(0.9)	< 0.001
Head circumference (cm)^a^	33.9	(1.1)	35.4	(1.2)	< 0.001
Length (cm)^b^	48.6	(2.0)	51.1	(1.9)	< 0.001
Ponderal index (g/cm^3^)^b^	2.5	(0.2)	2.8	(0.3)	< 0.001
Maternal age (years)	28.3	(3.5)	30.7	(4.4)	0.002
Parental SES, n (%)^c^					
SES class 1	1	(2.6)	1	(2.0)	
SES class 2	8	(20.5)	7	(13.7)	
SES class 3	7	(17.9)	13	(25.5)	0.518
SES class 4	14	(35.9)	14	(27.5)	
SES class 5	9	(23.1)	16	(31.4)	
Age at follow-up (years)	32.5	(0.6)	32.6	(0.5)	0.690

	n	(%)	n	(%)	
Women	22	(47.8)	35	(57.4)	0.327
Education at follow-up					
Lower secondary or lower (ISCED 1–2)	1	(2.2)	0	(0)	
Intermediate (ISCED 3–5)	19	(41.3)	22	(36.1)	0.444
Lower tertiary or higher (ISCED 6–8)	26	(56.5)	39	(63.9)	

*ISCED* International Standard Classification of Education, *SD* standard deviation, *SES* socioeconomic status (1–5, where 5 is highest), *SGA* small for gestational age.

^a^Data missing for five participants born SGA and four controls.

^b^Data missing for five participants born SGA and three controls.

^c^Data missing for seven participants born SGA and 10 controls.

p-values based on Student’s t-test for continuous data and Exact Mann–Whitney U test for ordinal data (i.e., SES and ISCED).

At follow-up, mean height was significantly lower in the SGA group compared with the control group. The other anthropometric measurements and bioelectrical impedance analysis measures did not differ between the groups (Table 2).

**Table 2:** Anthropometric measures and bioelectrical impedance analysis of adults born small for gestational age (SGA) and non-SGA (control) at term.

	SGA (n = 46)		Control (n = 61)		Mean difference	(95% CI)
	Mean	(SD)	Mean	(SD)		
Height (cm)	170.8	(9.4)	174.6	(9.8)	− 3.8	(− 7.5 to − 0.1)
Weight (kg)	74.3	(16.7)	76.5	(16.0)	− 2.1	(− 8.5 to 4.2)
Body mass index (kg/m^2^)	25.4	(4.9)	25.0	(4.5)	0.4	(− 1.4 to 2.2)
Waist circumference (cm)	85.8	(13.7)	84.7	(11.4)	1.1	(− 3.7 to 5.9)
Hip circumference (cm)	100.7	(7.7)	102.0	(8.5)	− 1.2	(− 4.4 to 1.9)
Waist-to-hip ratio	0.85	(0.08)	0.83	(0.06)	0.02	(− 0.01 to 0.05)
Fat (%)^a^	27.1	(8.7)	26.6	(8.5)	0.6	(− 2.8 to 4.0)
Fat mass (kg)^a^	20.8	(10.4)	20.7	(9.6)	0.1	(− 3.9 to 4.0)
Fat free mass (kg)^a^	54.0	(11.3)	55.7	(11.7)	− 1.6	(− 6.2 to 2.9)
Muscle mass (kg)^a^	26.2	(6.6)	26.9	(6.7)	− 0.7	(− 3.3 to 1.9)
Total body water (kg)^a^	39.7	(8.3)	41.1	(8.5)	− 1.3	(− 4.7 to 2.0)

*CI* confidence interval, *SD* standard deviation, *SGA* small for gestational age.

^a^Data missing for two participants born SGA and three controls due to pregnancy, uncertainty about pregnancy and a nerve stimulator implant to treat chronic pain.

The results of the physical fitness tests are shown in Table 3. There were no group differences in grip strength, modified push-up test or step test results. Mean differences ranged from 0.08 SD units for the step test to 0.32 SD units for the modified push-up test. Separate analyses by sex showed that men in the SGA group had significantly lower grip strength in both hands compared with men in the control group. The mean difference was − 4.8 kg (95% CI − 0.6 to − 9.0 dominant hand, 95% CI − 0.4 to − 9.2 non-dominant hand, p for interaction SGA × sex = 0.112 and 0.162, respectively).

**Table 3:** Physical fitness of adults born small for gestational age (SGA) and non-SGA (control) at term.

	SGA (n = 46)		Control (n = 61)		Mean difference	(95% CI)
	Mean	(SD)	Mean	(SD)		
Grip strength, dominant hand (kg)	37.0	(8.2)	38.5	(10.3)	− 1.5	(− 5.1 to 2.0)
Women^a^	31.0	(5.0)	31.8	(5.5)	− 0.8	(− 3.7 to 2.1)
Men	42.5	(6.4)	47.3	(8.1)	− 4.8	(− 9.0 to − 0.6)
Grip strength, non-dominant hand (kg)	34.2	(8.4)	35.7	(10.2)	− 1.6	(− 5.2 to 2.1)
Women	28.1	(4.9)	29.2	(5.5)	− 1.1	(− 4.0 to 1.7)
Men	39.7	(7.0)	44.5	(8.4)	− 4.8	(− 9.2 to − 0.4)
Number of push-ups in 40 s	11.1	(4.8)	9.6	(4.1)	1.5	(− 0.2 to 3.2)
Women^a^	9.5	(4.4)	8.1	(4.1)	1.4	(− 0.9 to 3.8)
Men	12.5	(4.7)	11.7	(3.0)	0.8	(− 1.4 to 3.1)
Heart rate after 4 min step test	155.3	(18.7)	154.5	(19.4)	1.5	(− 5.9 to 8.9)
Women	150.8	(17.1)	150.4	(18.9)	0.4	(− 9.6 to 10.4)
Men^b^	159.9	(19.5)	160.0	(19.1)	− 0.1	(− 11.3 to 11.2)

*CI* confidence interval, *SD* standard deviation, *SGA* small for gestational age.

^a^Data missing for one control due to a hand fracture.

^b^Data missing for two participants born SGA who could not complete the test.

Adults born SGA were shorter than controls (Table 2). Height was associated with grip strength (*r* = 0.722, p < 0.001 dominant hand, *r* = 0.711, p < 0.001 non-dominant hand). When we adjusted for height, the difference in maximal grip strength among men decreased to − 2.8 kg (95% CI − 1.7 to 7.3 dominant hand, 95% CI − 2.0 to 7.6 non-dominant hand).

Results were unchanged regarding anthropometric measures and body composition when we performed sensitivity analyses by excluding eight participants born SGA and five controls who were pregnant, had musculoskeletal diagnoses or other conditions affecting physical functioning. However, the SGA group performed 2.4 (95% CI 0.7 to 4.1) more push-ups than the control group.

## Discussion

In this study we found no differences in body composition or physical fitness between adults born SGA and the control group, measured by grip strength, a 40-s modified push-up test and a 4-min submaximal step test. However, men in the SGA group had significantly lower grip strength in both the dominant and non-dominant hand compared with men in the control group.

A strength of this study includes the prospective population-based design, where participants were recruited and followed from mid-pregnancy. At birth, SGA was defined as birth weight below the 10th percentile. This may also comprise individuals who are genetically small and not necessarily growth restricted. Additionally, the control group may comprise individuals who are growth restricted, but still have a birth weight above the 10th percentile. This could possibly contribute to smaller differences between the groups in this study. Nevertheless, the 10th percentile is a common cut-off used to identify SGA individuals^6^. At the 32-year follow-up, 56.3% of the invited were assessed clinically. This low participation rate can partly be explained by the data collection being carried out during the Covid-19 pandemic. Even though follow-up rates of 50–80% participation have been suggested to be acceptable in cohort studies^39^, individuals performing worse may have a stronger tendency to drop out^39,40^. This could have led to a selection bias toward physically fit participants. However, there were few differences in background variables between participants and non-participants, and assessment of physical fitness was only a part of a larger follow-up examination. Thus, it seems unlikely that the results were affected by selection bias. Nevertheless, the loss to follow-up limits the sample size and hence gave wider confidence intervals than would be expected with a larger sample size.

Another strength is the use of objective measurement tools to assess body composition and physical fitness, as self-reports may be biased by over- or underestimation^41^. Assessments were carried out in the same order for all participants by trained examiners blinded to birth weight groups. Bioelectrical impedance analysis by the Seca® mBCA 515 has shown to agree well with the accurate and precise DXA method^42–44^, which is considered the reference measurement for differentiating lean and fat tissues. Both grip strength measured by a dynamometer and the modified push-up test are reported to be valid instruments for assessing muscular fitness^33,45^. A limitation of the study was the measurement of cardiorespiratory fitness by a submaximal test with heart rate as the outcome, as heart rate is largely individual^46^, and a maximal exercise test measuring maximal oxygen uptake would evaluate cardiorespiratory fitness more accurately^16^. However, a submaximal test was considered more feasible in this study, as it is less time consuming and more comfortable for the participants.

In this study, participants born SGA were shorter than controls, which we have previously documented in adolescence and young adulthood^11,13,47^. These findings are in line with other studies of children, adolescents, and adults^7,10,48,49^. However, evidence regarding overweight and adiposity is conflicting. We did not find differences in BMI, waist circumference or waist-to-hip ratio between the groups, consistent with our previous report of similar body composition of participants born SGA and controls at 26 years of age, measured by DXA^13^. Other studies of term-born adults with a birth weight < 10th percentile have reported both similar BMI and waist-to-hip ratio^50^, lower weight and reduced lean body mass^51^, and a higher percentage body fat^12^ and total abdominal fat mass^50^ compared with a control group. However, these studies used different birth weight percentiles to define the control group, which may explain some of the discrepancy.

Our hypothesis that adults born SGA would display a lower fitness level than their peers was not confirmed in this study. However, men born SGA had approximately 5 kg lower grip strength than men in the control group in the unadjusted analyses. This result must be interpreted with caution, as the 95% CI was rather wide. The difference in grip strength is consistent with the recent Swedish study that found strong associations between birth weight in men born at term and grip strength at 18 years of age^27^. However, in that study a one SD lower birth weight was associated with 1.8 kg lower grip strength. In the present study, mean difference in birth weight SD score was 1.44, corresponding to a 2.6 kg difference. This is consistent with what we observed in men and would also be included in the CI we observed among women. Further, our finding is also in accordance with other studies that have found strong associations between lower birth weight and reduced grip strength in adulthood, regardless of gestational age at birth^21^. The reduced grip strength found for men born SGA in this study could indicate increased risk of negative health outcomes, as increased hazard ratio of all-cause mortality ranging from 1.08^20^ to 1.16^19^, and for cardiovascular mortality of 1.17^19^, have been reported for every 5 kg reduction in grip strength.

The differences in grip strength among men only may be related to motor development, as associations between motor development and grip strength in adulthood have been documented^22^. Growing up, boys in the general population are reported to have motor problems more often than girls^52^. In the SGA population, several studies also show that boys are more vulnerable to growth restriction in utero than girls, possibly because of a higher growth velocity^3^. Thus, boys and men born SGA may be more susceptible to unfavourable development outcomes. In support of this, we have previously reported reduced manual dexterity at 14 years of age in boys born SGA, and not girls^47^. This may be related to the findings of reduced grip strength in the present study. When we adjusted for height, the difference in grip strength was reduced and no longer significant, indicating that the difference was partly mediated through a lower height in men born SGA. This is in accordance with previous research reporting that height is associated with grip strength^22^. However, when grip strength is used as a predictor of mortality and functional capacity it is not adjusted for height^19,20,53^. Additionally, even when adjusted for height, the grip strength of men born SGA was lower than the 59 kg normative value for men of the same age in Norway^35,54^. This underlines the relevance of the lower grip strength finding in our study.

Contrary to our hypothesis, we did not find any differences between the groups in the push-up test or step test. In a sensitivity analysis excluding participants with conditions affecting physical functioning, the adults born SGA even performed more push-ups than the controls. In a study of 287,000 male military conscripts Ahlquist et al.^26^ reported that among term-born men, each unit decrease in birth weight z-score was associated with reduced cardiorespiratory fitness of 7.9 W in maximal workload, corresponding to approximately 0.2 SD in that population. That study did not compare men born SGA with men born non-SGA and used a different proxy for cardiorespiratory fitness than we did. However, our confidence intervals among men ranged from less than − 0.5 SD to more than + 0.5 SD, and we may not have had adequate power to observe an association that was observed in the paper of Ahlquist et al.^26^. Ridgway et al.^22^ reported a weak association between lower birth weight and lower aerobic fitness, however, the sample also included late preterm born individuals. Our results are consistent with our previous findings at 18 years of age^11^ and with two other small studies of Danish men that found no differences in VO_2max_ between those with birth weight ≤ 10th percentile and a control group at 19 and 24 years of age^24,25^. Thus, it seems unlikely that adults born SGA have any moderate or large deficit in cardiorespiratory fitness, but we cannot exclude a weak association with lower birth weight.

Overall, the lack of differences between adults born SGA and non-SGA controls in this study is promising with regards to future health. However, reduced grip strength is an established predictor of future physical function, morbidity and mortality^19,20,53^, and is shown to track through life^53^. It is therefore worrying that men born SGA already at 32 years of age had reduced grip strength compared with men in the control group. Consequently, promoting a physically active lifestyle in men born SGA may be advantageous. A physically active lifestyle has been shown to form early in life^55^, indicating that promotion of physical activity, especially concerning activities that enhance muscular strength, should be a focus from childhood.

## Conclusion

Overall, we found no differences in body composition or physical fitness between adults born SGA and non-SGA at term. However, men born SGA had lower grip strength than men born non-SGA. There are few studies concerning physical fitness in individuals born SGA at term entering mid-adulthood. Further research is therefore needed to determine whether adults born SGA have lower physical fitness than their non-SGA peers.

## Data Availability

The datasets generated and/or analysed during the current study are not publicly available because permission has not been applied for from neither the participants nor the Ethical Committee but are available from the corresponding author on reasonable request.
